# Chicken antibodies are highly suitable for particle enhanced turbidimetric assays

**DOI:** 10.3389/fimmu.2022.1016781

**Published:** 2022-10-11

**Authors:** Anders Larsson, Andrew Campbell, Mats Eriksson

**Affiliations:** ^1^ Department of Medical Sciences, Uppsala University, Uppsala University Hospital, Uppsala, Sweden; ^2^ NABAS AS, Ås, Norway; ^3^ Department of Surgical Sciences, Section of Anaesthesiology and Intensive Care Medicine, Uppsala University, Uppsala, Sweden; ^4^ NOVA Medical School, New University of Lisbon, Lisboa, Portugal

**Keywords:** chicken IgY, cystatin C, free light chains, immunoassays, particle enhanced turbidimetric assay, PETIA

## Abstract

Antibody-based assays are commonly used in clinical laboratories for analyzing plasma, serum and other samples for particular protein markers. Although such assays have been traditionally based on antibodies raised in mammals (e.g., mice, rabbits, goats), there are several advantages of using avian antibodies (IgY) raised in chickens, including production volumes, costs, and ethical/animal welfare considerations. A further disadvantage of using mammalian IgG in such assays is the potential for agglutination when exposed to rheumatoid factor (RF) in serum. However, when used in the free form the immune complexes formed with avian antibodies have been reported to have less ability than those formed with mammalian antibodies to cause the light scatter which are used for instrument measurement. In addition, when the amount of antigen exceeds the maximum precipitating point in relation to the amount of antibody, there is a rapid decline in the absorbance values of the immune complexes (antigen excess) when IgY is used. However, when avian antibodies are conjugated to a substrate and used in particle enhanced turbidimetric assays (PETIA), these problems are avoided. Here we investigated three clinical assays using chicken antibodies, one using free (unbound) IgY and two with IgY-based PETIA. The IgY PETIA demonstrated a strong scatter response, even at high antigen concentrations in contrast to the steep decline seen with free IgY antibodies. IgY PETIA reagents can provide test results with low coefficient of variation (<1% for duplicate samples). We also investigated the effect of RF on agglutination of mammalian antibodies (IgG from mouse, rabbit, sheep, and human) and chicken antibodies. Whereas agglutination was observed with all the mammalian antibodies in the presence of RF, this was not observed at all with chicken IgY. Our results support the growing body of evidence that chicken egg yolks can thus be a valuable source of antibodies for use in PETIA in clinical laboratories.

## Introduction

Turbidimetric and nephelometric assays are widely used in clinical laboratories for quantifying abundant plasma proteins. Turbidimetric methods can easily be applied to the large chemistry analyzers used in such laboratories. These instruments are highly automated and have a capacity of 1000 assays or more per hour. The platforms usually have a broad assay panel (>30 different assays) and are run 24/7. The methods have assay times of around 10 min and measure changes in absorbance due to the scattered light that occurs when an antigen combines with an antibody to form complexes, enabling rapid reporting of patient test results. In most laboratories, including Uppsala University Hospital, Sweden, the test results are reported back to the patients’ electronic files within 40 min after the samples arrive at the laboratory. The initially developed assays used free (unbound) antibodies, often with polyethylene glycol to enhance the precipitation reaction. These assays measure proteins from approximately 0.1 g/L and higher. Assays that use free antibodies quantify, for example, serum/plasma albumin, IgG, IgA, IgM, haptoglobin, fibrinogen, transferrin, antitrypsin, and alpha-acid-glycoprotein. Each of these markers represent at least 100,000 test results per year in Sweden (1).

If the antibodies are coupled to particles (particle enhanced turbidimetric immunoassays; PETIA) (2, 3), rather than being free, the reaction is amplified, and the technology can be used down to concentrations of approximately 0.1 mg/L of analyte. The particles used are latex-based and the antibodies are coupled to the surface. Typical PETIAs measure urine albumin, plasma C-reactive protein, ferritin, soluble transferrin receptor, cystatin C, immunoglobulin free light chains, cerebrospinal fluid IgG, and albumin. Several million PETIAs are performed annually in Sweden (1). The methodology requires more antibodies per test than, for example, a sandwich enzyme-linked immunosorbent assay (ELISA), and the large number of assays means that the total antibody volumes required are much higher than for ELISA methods.

Antibodies for such tests are usually raised in mammals such as mice, rabbits, or goats. However, antibodies for these tests could also be raised in birds. Production of large volumes of antibodies is one of the strengths of using yolk antibodies raised in chickens. One rabbit has been reported to produce 150 mg antibody as compared to 15 g produced by one chicken (4).

Other advantages with using avian antibodies are the lack of interference due to rheumatoid factor (RF) or anti-mammalian IgG antibodies (5, 6). The most well-known of these anti-mammalian IgG antibodies is probably human anti-mouse IgG antibodies (HAMA) (7).

The perspective of animal welfare should not be overlooked in this context. The effectiveness of isolation of IgY from egg yolk enables fewer number of animals to be used, and there is no requirement for blood sampling. This is in agreement with the recommendations of the European Centre for the Validation of Alternative Methods (ECVAM), recommending that egg yolk antibodies should be used instead of mammalian antibodies (8).

The most widely used IgY based PETIAs are for human cystatin C, human fecal calprotectin, human serum/plasma calprotectin and dog CRP. Cystatin C is a low molecular weight protein that is produced by all cells in the body and is removed from the circulation by glomerular filtration in the kidneys. Cystatin C is widely used as an alternative to creatinine to evaluate kidney glomerular filtration rate (GFR). The IgY PETIA is one of the most frequently used serum/plasma cystatin C assays. It has been shown to be superior to creatinine for the prediction of kidney function and as a cardiovascular risk marker (9–13). The IgY PETIA has also been used to detect cystatin C in urine (14), seminal plasma (15) and cerebrospinal fluid (16).

Calprotectin is the most abundant protein in the cytoplasma of neutrophils and it is used as a marker for neutrophil activation. Elevated fecal calprotectin is a marker for migration of neutrophils to the intestinal mucosa, which is caused by inflammatory bowel disease. Fecal calprotectin is today widely used for the detection of inflammatory bowel diseases (17–19). One of the most frequently used tests is fCal Turbo which is an IgY based PETIA (20–23). The assay has also been used for the detection of fecal calprotectin in dogs and cats (24).

The IgY PETIA for serum/plasma calprotectin has been used as a marker for inflammatory conditions including bacterial infections such as sepsis (25, 26). It has been shown to be a superior sepsis and infectious disease marker to e.g. procalcitonin which is a widely used marker for bacterial infections (27–31).

There is also a IgY PETIA specific for dog CRP (32, 33). CRP is a valuable inflammation marker in humans. The crossreactivity between human and dog CRP varies. Thus, veterinarians screened human CRP reagent lots to find a lot that showed a strong crossreactivity to dog CRP and used that lot until it expired and then they searched for a new lot. The development of the specific dog CRP PETIA ensured a better lot to lot consistency and eliminated the search process to find suitable lots.

This report describes the use of avian antibodies in PETIA methods that are suitable for routine laboratory assays.

## Materials and methods

### Turbidimetric assays

Three different turbidimetric assays in which IgY-based immunoassays were used to assess the relative content of specific analyte antigens are summarized below. See **Table 1** for an overview of the assay parameters. The samples used were Li-heparin samples sent to the Department of Clinical Chemistry and Pharmacology. The blood samples were collected in vacutainer plasma separation tubes. The use of surplus patient samples without patient identities was approved by the Uppsala University Ethical committee (approval 01/367).

**Table 1:** Overview of assays.

	Assay 1	Assay 2	Assay 3
**Analyte**	Fibrinogen	Kappa free light chains	cystatin C
**Material**	Plasma	Calibrator	Plasma
**Antibody**	Chicken IgY	Chicken IgY	Chicken IgY
**Free antibodies or PETIA**	Free antibodies	PETIA	PETIA
**Analysis platform**	Beckman Array System	Alcor Analyzer	Architect c8000 Analyzer

#### Assay 1: turbidimetric assay using unbound chicken IgY antibodies for fibrinogen

The reaction between free chicken anti-fibrinogen antibodies (IgY Lab Systems, Uppsala, Sweden) and fibrinogen in plasma samples was analyzed using a Beckman Array system (Beckman Coulter, Brea, CA, USA), with 42 µl of samples prediluted in 0.9% NaCl added to the measuring cuvette. The avian anti-fibrinogen antibody vial was placed in one of the instrument’s antibody reagent positions and the assay was performed with the buffers and settings provided by Beckman Coulter.

#### Assay 2: PETIA using chicken IgY for kappa immunoglobulin free light chains

IgY Production: Chicken antibodies specific for human kappa free light chain (FLC) were generated at NABAS AS (ÅS, Norway) by immunizing chickens with kappa FLC that had been purified from urine samples containing Bence Jones (BJ) proteins (kindly provided by A. Grubb, Lund University Hospital, Sweden). IgY was isolated from egg yolks using the water dilution method of Akita & Nakai (34), followed by salt precipitation. It was then affinity purified using the kappa FLC antigen immobilized onto HiTrap NHS-activated crosslinked agarose (Cytiva 17-0716-01), and adsorbed free of whole molecule kappa light chains, in a procedure as described by Bradwell et al. (35).

PETIA reagent 1 (R1): Kappa FLC antibodies were covalently conjugated to 194 nm latex particles (IKERLAT Polymers S.L., Gipuzkoa, Spain) and stabilized in a 25 mM tris buffer, pH 8.2. The PETIA reaction buffer, reagent 2 (R2), was a 3-(N-morpholino) propanesulfonic acid buffer, pH 6.9.

Kappa FLC measurements were performed on an Alcor analyser (Edif Instruments s.r.l., Italy) using the following instrument settings: primary wavelength 600 nm; 180 µl R1 and 3 µl sample mixed with 60 µl R2, and the endpoint measurement recorded after 544 seconds (just under 10 minutes).

A single 9-point calibration curve (range 0 – 231 mg/L) was prepared using serial dilutions of a purified polyclonal kappa FLC antigen isolated from pooled human urine (Advy Chemical Pvt Mumbai, India). Kappa FLC values were assigned from measurements obtained using the Binding Site (The Binding Site Group, Birmingham, UK) kappa FLC assay.

#### Assay 3: PETIA using chicken IgY for cystatin C

The cystatin C method was introduced as a routine method at the Department of Clinical Chemistry and Pharmacology in 2007. Since the introduction we have produced close to one million test results using the IgY based PETIA. Immunoparticles coated with purified chicken antibodies to cystatin C were obtained from Gentian AS (Moss, Norway). Plasma cystatin-C measurements were performed on an Architect c8000 analyzer (Abbott Laboratories, Abbott Park, IL, USA) using the following instrument settings: primary wavelength 548 nm, 7-point spline calibration; 220 µl reagent 1 (R1), 3 µl plasma sample, and 45 µl reagent 2 (R2). The analyzer measured the turbidimetry in the samples every eighteenth second. At time zero, the buffer was added (R1) and then the plasma samples were added to the R1 and mixed before adding the immunoparticles (R2) at reading point 16 and recording the sample blank at reading point 18. The increase in absorbance was calculated as endpoint readings (difference between results at reading points 18 and 33) or as rate readings. A rate reading was defined as the steepest slope value around time points 19 to 24. The total assay time is 33 x 18 seconds (c.a. 10 min). The measuring range for the cystatin C method was 0.3-8.1 mg/L. Assay precision was investigated for both rate and endpoint measurements by calculating the coefficient of variation (%CV) from duplicate measurements.

#### The effect of rheumatoid factor on immunoparticle agglutination when using mammalian or avian antibodies

Immunoparticles were coated with various purified antibodies: chicken IgY, and IgG from mouse, rabbit, sheep, and human using standard methods. The effect of RF on the particles was investigated by mixing 20 µl of the different coated particles with samples of serum known to contain RF from ten patients and with serum samples from ten healthy blood donors. The serum was diluted 1:4 in saline prior to investigation. Agglutination of the particles was determined visually.

## Results

### Turbidimetric assays

#### Assay 1: turbidimetric assay using unbound chicken IgY antibodies for fibrinogen

**Figure 1:**
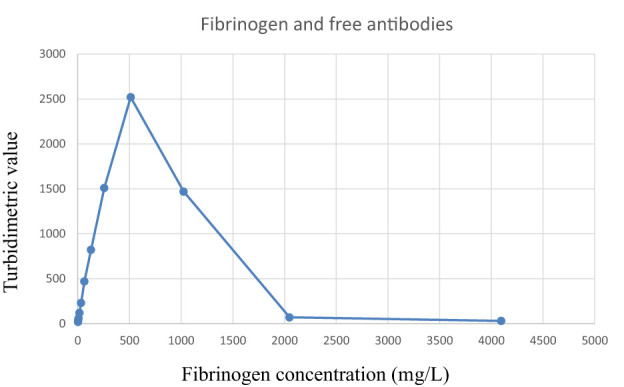
Reaction curve when using free avian anti-fibrinogen antibodies to detect fibrinogen in plasma samples. Observe the rapid decrease when the fibrinogen concentrations exceed the precipitation maximum (right hand side of the plot). The samples were analyzed as singletons.

The absorbance increased with increasing amounts of fibrinogen in the samples until it reached a peak around 500 mg/L. After the peak, there was a very rapid decline in absorbance as the amount of antigen further increased (**Figure 1**).

#### Assay 2: PETIA using chicken IgY for kappa immunoglobulin free light chains

**Figure 2:**
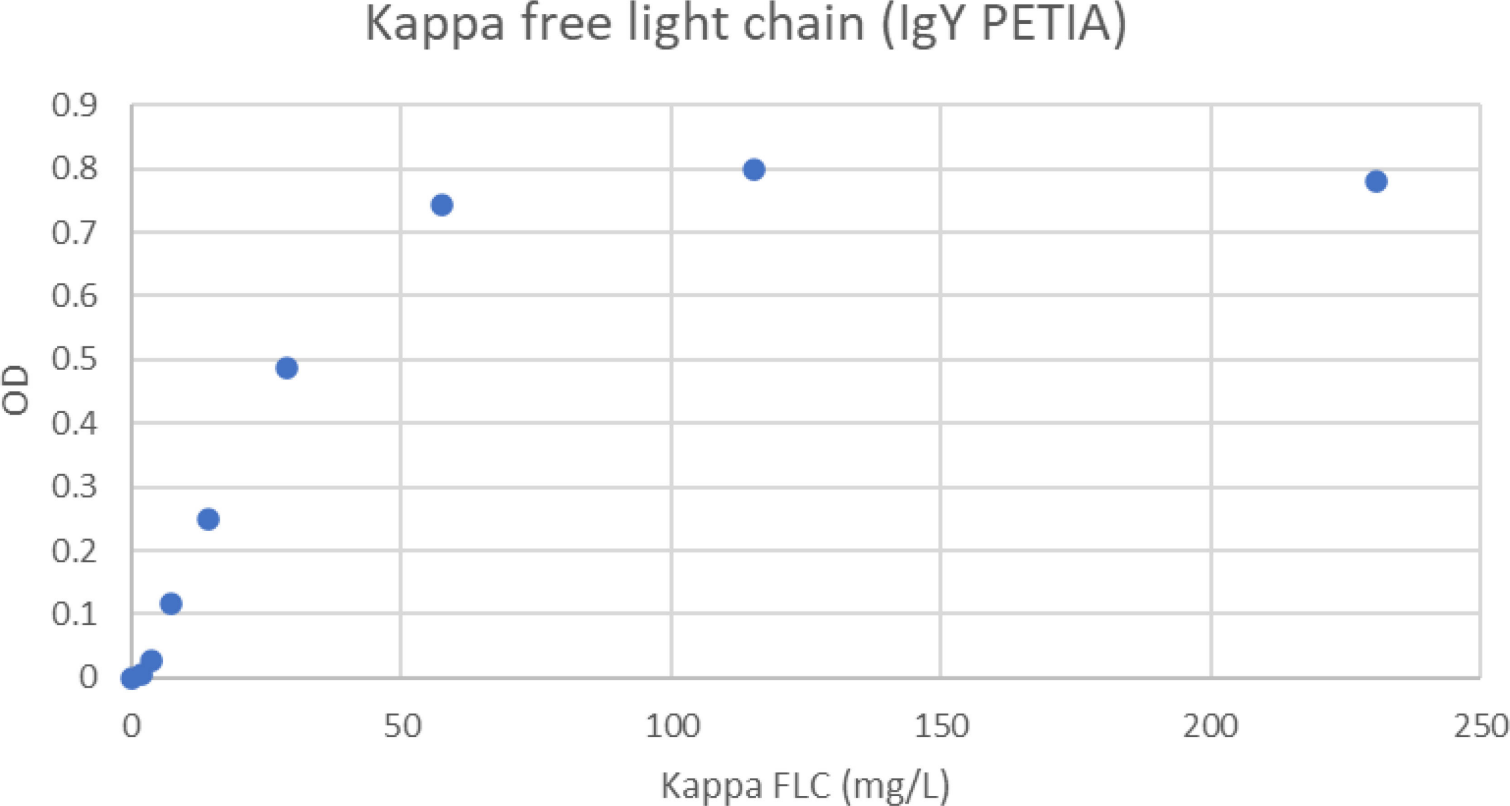
Antigen excess curve for kappa FLC PETIA. The figure shows the free kappa chain concentration in the samples versus the absorbance readings. The samples were analyzed as singletons. The plot shows that antigen excess is not present below 230 mg/L free kappa chain.

When the concentration of kappa FLC increased in the samples, an antigen excess situation was reached, and the absorbance values started to decrease. Although kappa FLC values were in excess of 200 mg/L, there was still a strong reaction in excess of OD 0.78 (**Figure 2**).

#### Assay 3: PETIA using chicken IgY for cystatin C

**Figure 3:**
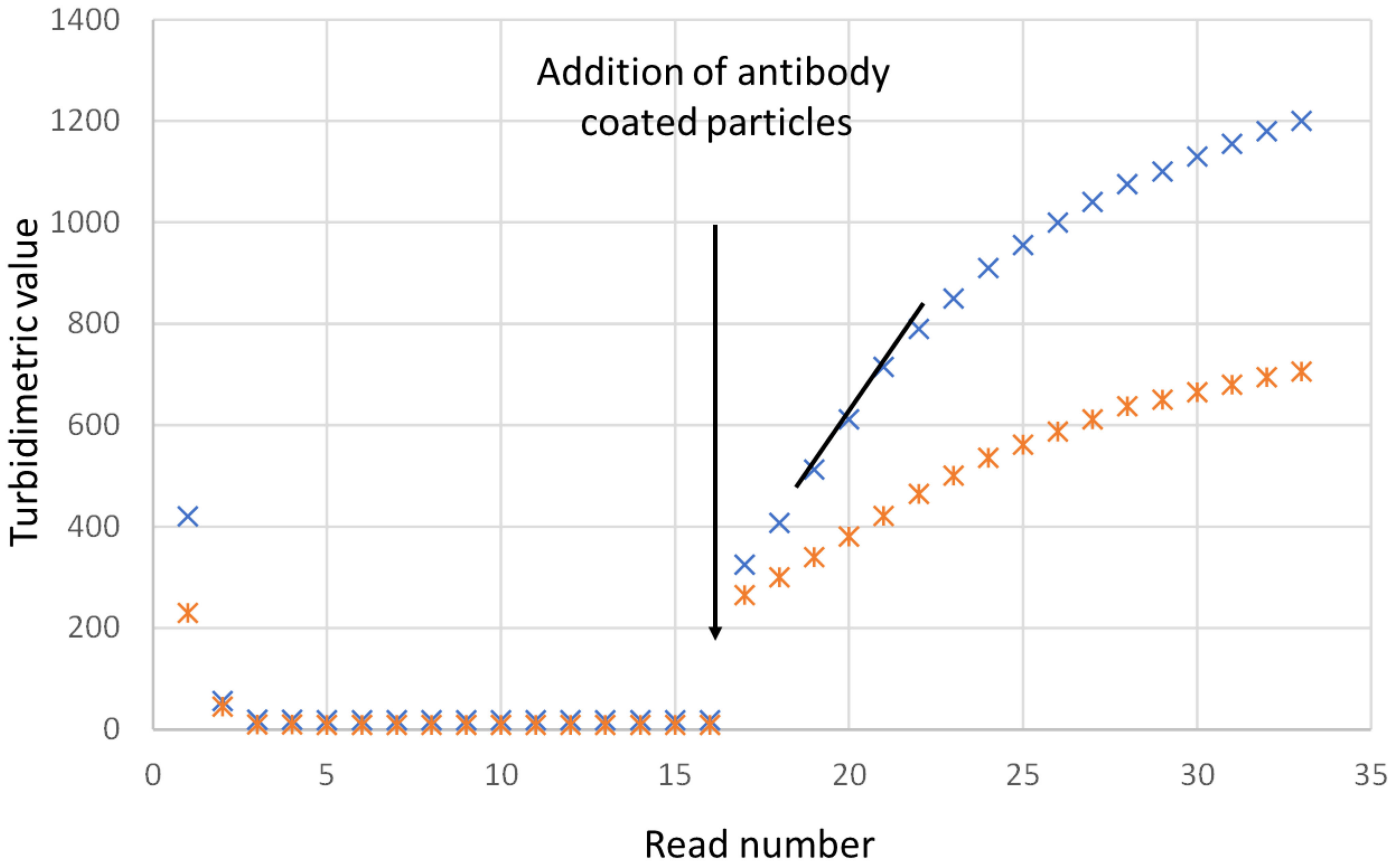
Presentation of the turbidimetric readings when analyzing cystatin C on an Architect c8000. The samples were analyzed as singletons. The instrument measures the turbidimetric readings every eighteenth second. The readings are shown for two samples, one sample with a cystatin C concentration of 7 mg/L (upper curve) and one sample with a cystatin C concentration of 3.5 mg/L. The antibody coated particles are added after position 16. The reaction between the particles and the sample can be measured as endpoint or rate readings. Rate is the steepest slope of the readings after position 18 and is represented in the figure as a black line.

**Figure 3** shows typical turbidimetric progress curves for the IgY cystatin C PETIA on the Architect c8000. The increase in turbidimetric absorbance is an effect of the amount of antigen in the sample and the concentration of cystatin C was calculated using the standard curve generated using the calibrator set.

The mean %CV of the calculated cystatin C (in mg/L) for duplicate samples was 0.58% (n=179) for endpoint measurements and 0.50% (n=118) for rate determinations.

#### The effect of rheumatoid factor on immunoparticle agglutination when using mammalian or avian antibodies

**Figure 4:**
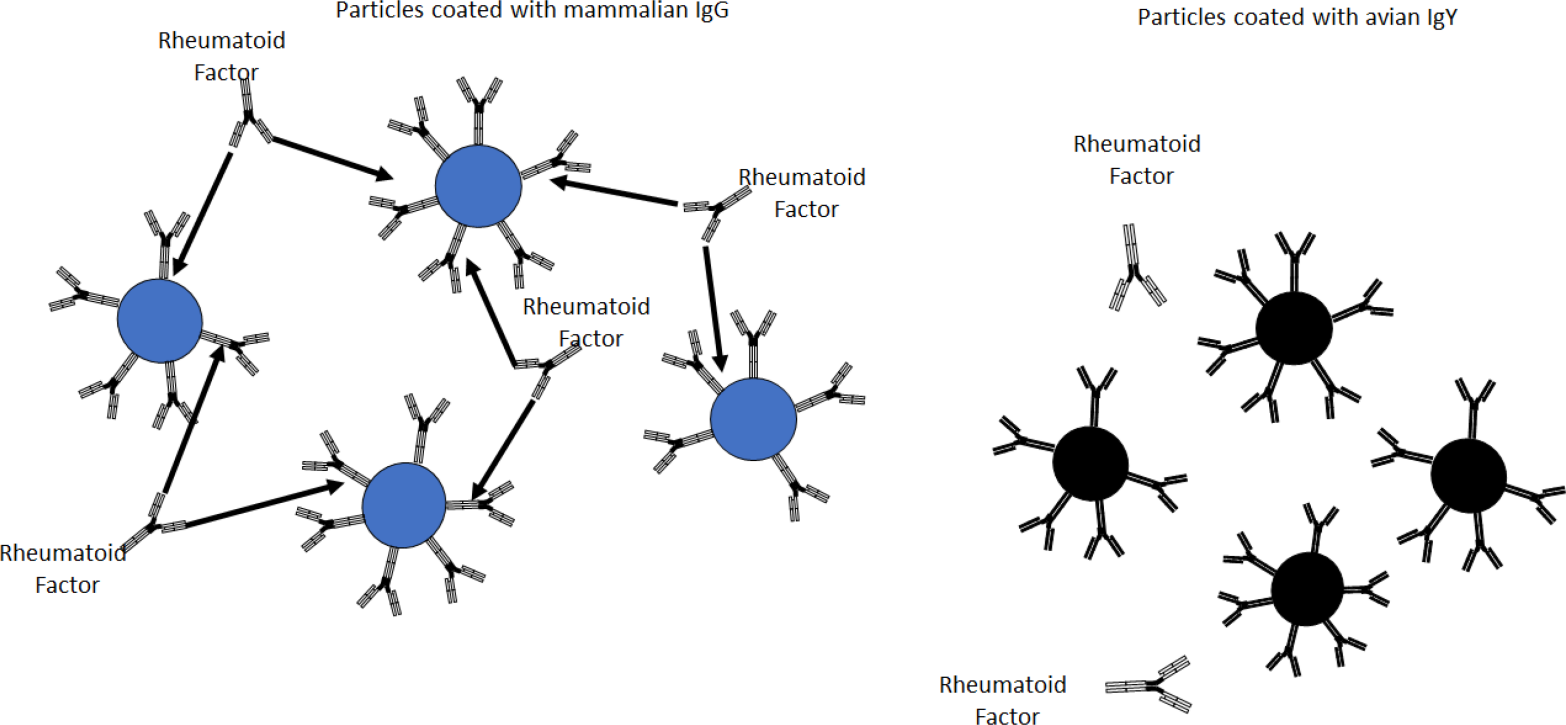
Rheumatoid factor-positive sera agglutinated particles coated with mammalian IgG (left) but not particles coated with chicken IgY (right).

All immunoparticles coated with mouse, rabbit, sheep, and human IgG agglutinated when mixed with RF-positive sera (**Figure 4**). The immunoparticles coated with chicken IgY did not agglutinate when mixed with RF-positive sera. None of the particles agglutinated when mixed with the blood donor samples, regardless of the type and source of antibody.

## Discussion

The main finding from this study was that whereas there is a rapid decrease in absorbance when reaching the antigen excess area in assays using avian antibodies in solution, for IgY PETIAs there is a very favorable antigen excess curve. In addition, IgY-based PETIA reagents demonstrate high absorbance values and high precision (low coefficients of variation).

There are several benefits from using IgY purified from egg yolk in clinical PETIA: firstly, purification is simple and relatively inexpensive (36); secondly, there is a cost-effective perspective on IgY, as one hen may produce up to a quantity of 2 g of IgY monthly in egg yolk, which corresponds to approximately 600 ml of whole blood; more than 100 mg of purified IgY may be isolated from just one egg (37); thirdly, the immunological response is more potent with increasing phylogenetic distance between the antigen source and the responding immune system (38–40). Thus, the greater evolutionary distance provides an advantage in using IgY rather than IgG as the primary antibody in some immunoassays. Using IgY, means that the risk of IgG binding to the antigenic site of an epitope, identified through a cross-reaction with a secondary mammalian antibody, minimized (41, 42). Furthermore, chicken IgY does not cross-react with mammalian IgG, but mammalian epitopes are recognized more readily by chicken IgY than by mammalian antibodies (41, 43, 44).

Polyclonal IgY antibodies have a very good storage stability. When IgY antibodies, originating from egg yolk, were stored at + 4°C in saline buffered with phosphate and mixed with 0.02% NaN3, there was no significant loss of antibody titer after 30 years of storage. The activity of affinity-purified and biotinylated IgY antibodies of avian origin was well preserved after 5 years of storage at + 4°C (7). After 6 months of storage the paratope of these purified antibodies was adequately retained.

Chicken IgY is thus an interesting alternative for large-scale production of antibodies for diagnostic purposes. As with mammalian IgG antibodies, IgY has two antigen-binding sites and should precipitate multivalent antigens. Even in the presence of polyethylene glycol 6000, chicken antibodies form antibody-antigen complexes that result in less scatter than a corresponding complex containing mammalian antibodies. Chicken antibodies lack the hinge region of mammalian antibodies and are thus more rigid. This possibly contributes to the lower light-scattering ability of the complexes when chicken antibodies are used free (not bound to particles). Another aspect to consider is that, in the unbound form, avian antibodies have a much steeper decline in the antigen-excess part of the precipitation curve. In principle, sample results should all be on the increasing part of the precipitation curve (left-hand side). If the concentration of analyte in a patient sample is so high that the result is on the right-hand side of the curve, then the result will be similar to that from a sample with a value on the left-hand side, and the instrument will report the lower value. Biomarkers for which patients may have very high values are, for instance, tumor markers, IgG, IgA, IgM, FLC (myeloma patients), and urine albumin. To avoid erroneous results, the methods have a safety margin that ensures that a test result does not surpass the peak value of the precipitation curve. The rapid decline when free (unbound) antibodies are used means that this safety margin is narrow. In order to increase the safety margin, the amounts of antibodies need to be increased. The problems associated with antigen excess have been particularly noted for FLC assays (45). When using IgY, the assay can be used with an automatic rerun at higher sample dilutions when the absorbance value is above 0.75. This will give a good safety margin and minimize the risk of erroneously reporting low values for antigen-excess samples. However, when avian antibodies are used in PETIA, they behave very differently to free antibodies; they give higher turbidimetric readings and have a broader antigen-excess margin. As IgY PETIA assays have a very slow decline from peak value, thereby providing a much broader safety margin, this is clearly a better format for such assays.

Chicken IgY have several biochemical advantages when used for the detection of mammalian or bacterial antigens. Egg yolk antibodies could thus replace mammalian serum antibodies in many immunoassays. Still there is a very limited use of avian antibodies. This is most likely due to tradition. There are a lot more rabbit or mouse antibodies commercially available that can be combined with secondary antibodies labelled with a broad range of detector molecules. The number of publications on the use of chicken antibodies is much more limited compared to mammalian antibodies.

One explanation could be that most researchers are used to bleeding rabbits and handling serum samples. It is much more difficult to draw blood from a hen or a rooster. Chickens have very fragile veins, so a venous puncture usually results in a large haemorrhage in the subcutaneous tissue which blocks the view. Also, chicken have very limited clot retraction. The amount of serum is therefore usually less than 10% of the total blood volume. This can be circumvented by adding an anticoagulant and collect plasma instead of serum. Most researchers are not familiar with the use of yolk as a source of antibodies. The purification of yolk antibodies requires different methodologies to the purification of mammalian antibodies from serum. Other obstacles are that commonly used methods for purification of mammalian antibodies such as protein A and protein G can not be used for purification of avian antibodies.

We have previously shown that IgY-based PETIA reagents gave a stronger turbidimetric reaction than rabbit antibody-based PETIA reagents (46). The strong reaction is probably an important factor for the low CV obtained with the IgY-based PETIA for analyzing for cystatin C. Despite the low plasma sample volumes (3 µl) used in the current study, we observed %CVs of 0.58% for endpoint measurements and 0.50% for rate determinations. The CV for rate measurements is lower than for endpoint measurements. Thus, it is not certain that the best results are always obtained with rate measurements.

Yolk antibodies are usually pooled over time and from several animals. This should, theoretically, reduce batch-to-batch variation in the antibodies produced. In our experience, batch-to-batch variation of the cystatin C immunoparticles was below 3% over several years (47). Furthermore, storage of eggs at +4°C for up to 180 days did not significantly lower antibody activity in egg yolk as compared to fresh eggs (48).

The lack of reactivity between RF positive sera and IgY-coated immunoparticles observed in our study is supported by previous publications reporting that neither RF nor human anti-mouse IgG antibodies do not react with avian antibodies (7, 49).

In conclusion, using IgY in PETIA assays provides stable results (very low %CV), and IgY-PETIA are preferable to free-IgY immunoprecipitation methods when the analyte antigen is in excess.

Thus, IgY is very well suited to use in PETIA and has several advantages over assays based on mammalian antibodies with respect to performance, RF reactivity, economic factors, and animal welfare considerations.

## Data availability statement

The original contributions presented in the study are included in the article/ supplementary material. Further inquiries can be directed to the corresponding author.

## Ethics statement

The studies involving human participants were reviewed and approved by the ethical committee at Uppsala University (01–367). The patients/participants provided their written informed consent to participate in this study.

## Author contributions

AL and ME conceptualized the study. AL and AC designed the experiments, collated the assay results, and performed the data analysis. AL, AC, and ME wrote the manuscript. All authors contributed to the article and approved the submitted version.

## Funding

The project was supported by funding from the Uppsala University Research Fund. The kappa free light chain study was supported by Eurostars (grant number 114334).

## Acknowledgments

We are grateful to scientific staff at NABAS and Uppsala University Hospital for assistance in running the assays. Lucy J. Robertson is acknowledged for critical comment and revision in preparation of this manuscript.

## Conflict of interest

AC is an employee of NABAS, a chicken IgY manufacturer and supplier, and AL is a board member of IgY Lab Systems AB, a chicken IgY manufacturer.

The remaining author declares that the research was conducted in the absence of any commercial or financial relationships that could be construed as a potential conflict of interest.

## Publisher’s note

All claims expressed in this article are solely those of the authors and do not necessarily represent those of their affiliated organizations, or those of the publisher, the editors and the reviewers. Any product that may be evaluated in this article, or claim that may be made by its manufacturer, is not guaranteed or endorsed by the publisher.
